# Identification of TLR2 as a Key Target in Neuroinflammation in Vascular Dementia

**DOI:** 10.3389/fgene.2022.860122

**Published:** 2022-07-06

**Authors:** Yuye Wang, Shuang Lv, Xiao Zhou, Xiaoqian Niu, Leian Chen, Ziyuan Yang, Dantao Peng

**Affiliations:** ^1^ Department of Neurology, China-Japan Friendship Hospital, Beijing, China; ^2^ Graduate School of Peking Union Medical College and Chinese Academy of Medical Sciences, Beijing, China; ^3^ Peking University China-Japan Friendship School of Clinical Medicine, Beijing, China

**Keywords:** vascular dementia, TLR2, neuroinflammation, bioinformatic analysis, WGCNA

## Abstract

Vascular dementia (VaD) is the second most common cause of dementia. At present, precise molecular processes of VaD are unclear. We attempted to discover the VaD relevant candidate genes, enrichment biological processes and pathways, key targets, and the underlying mechanism by microarray bioinformatic analysis. We selected GSE122063 related to the autopsy samples of VaD for analysis. We first took use of Weighted Gene Co-expression Network Analysis (WGCNA) to achieve modules related to VaD and hub genes. Second, we filtered out significant differentially expressed genes (DEGs). Third, significant DEGs then went through Geno Ontology and Kyoto Encyclopedia of Genes and Genomes (KEGG) analysis. Fourth, Gene Set Enrichment Analysis (GSEA) was performed. At last, we constructed the protein–protein interaction (PPI) network. The results showed that the yellow module had the strongest correlation with VaD, and we finally identified 21 hub genes. Toll-like receptor 2 (TLR2) was the top hub gene and was strongly correlated with other possible candidate genes. In total, 456 significant DEGs were filtered out and these genes were found to be enriched in the Toll receptor signaling pathway and several other immune-related pathways. In addition, Gene Set Enrichment Analysis results showed that similar pathways were significantly over-represented in TLR2-high samples. In the PPI network, TLR2 was still an important node with high weight and combined scores. We concluded that the TLR2 acts as a key target in neuroinflammation which may participate in the pathophysiological process of VaD.

## Introduction

Vascular dementia (VaD), following Alzheimer’s disease (AD), is one of the most prevalent causes of dementia (O'Brien and Thomas, 2015). A study in 6,481 Korean older adults showed that in 2016 disability-adjusted life-years (DALYs) caused by VaD (316 per 100,000) comprised 20% of the total DALYs caused by mild cognitive impairment (MCI) and dementia. In 2065, DALYs due to VaD (3654 per 100,000) would comprise 38% of the total DALYs as mentioned before. In parallel, the years of life lived with disability (YLDs) attributed to VaD (85 per 100,000) accounted for 18% of the total YLDs caused by MCI and dementia in 2016, while in 2065 YLDs attributed to VaD (410 per 100,000) will account for 15% of total YLDs (Moon et al., 2021). As the data shows, DALYs and YLDs of VaD are estimated to increase. However, there are fewer relative studies about VaD than those about AD, and there are no licensed treatments for VaD.

As a multifactorial disease, various risk factors participate in the development of VaD. Age and stroke are both major risk factors for the pathogenesis of VaD. VaD is also associated with vascular risk factors (O'Brien and Thomas, 2015; Iadecola et al., 2019). In addition, genetic linkage analyses investigated penetrant monogenic causes of VaD (Romay et al., 2019). Thus, a comprehensive understanding of key risk factors and genetic predispositions that lead to VaD needs to be clarified.

In nervous system, Toll-like receptors (TLRs) were reported to regulate the numbers of neurons and the size of brain, modulating structural plasticity in the adult brain (Li G et al., 2020). TLRs were an ancient family of pattern recognition receptors (PRRs). The role of TLRs in immunity control has been broadly discussed (Fitzgerald and Kagan, 2020). In neurological diseases, TLRs were reported to participate in AD (7), Parkinson’s disease (PD) (Kouli et al., 2019), ischemic stroke (IS) (Wang et al., 2013; Tajalli-Nezhad et al., 2019), and multiple sclerosis (MS) (Racke and Drew, 2009). However, the role of TLRs in VaD remained unclear.

In the present study, we performed a bioinformatic analysis based on GSE122063 (McKay et al., 2019). We first tried to figure out hub genes and top hub gene. Then we conducted a basic analysis on DEGs. Last, we performed relative analyses centered on the top hub gene to further investigate the probable mechanism of that gene in VaD.

## Materials and Methods

### Microarray Data Processing

In the Gene Expression Omnibus (GEO, https://www.ncbi.nlm.nih.gov/geo/) database, we chose GSE122063 which included the autopsy samples of VaD for analysis. GSE122063 was based on GPL16699 which used Agilent-039494 SurePrint G3 Human GE v2 8 × 60 K Microarray to detect the expression of genes. The microarray data includes eight VaD patients, 12 AD patients, and 11 controls postmortem frontal and temporal cortex samples. Each sample was run with at least two technical replicates. Data from AD patients were excluded from analysis and VaD sample 1063 was removed due to poor data quality according to the clustering result. The raw expression matrix was directly downloaded from the website, and the SOFT format file was downloaded and parsed by the GEOquery package (Davis and Meltzer, 2007). Then we used GPL1699 to transit ID into gene names and gene symbols using merge function in R. In addition, we checked if the data need log transformation or normalization. After pre-processing, a normalized expression matrix was constructed. The group matrix was constructed based on clinical information. All bioinformatic analyses and visualization were processed based on R.

### Weighted Gene Co-Expression Network Analysis (WGCNA)

The WGCNA package (Langfelder and Horvath, 2008) was used to create a gene co-expression network. By median absolute deviation (MAD), the top 5,000 ranking genes were selected at first. Then a soft-thresholding power β was calculated by using the “pickSoftThreshold” function. A suitable power value was defined as the first number reaching which the degree of independence was at least 0.9. The gene expression matrix was then converted into a topological overlap matrix (TOM), and the genes were divided into several gene modules, each represented by a distinct color. Next, a hierarchical clustering analysis was performed by using the hclust function. Except for the WGCNA package, the gplots package (Warnes et al., 2020) was used for visualization. In addition, the top 100 networks sorted by weight were exported to Cytoscape software for visualization.

In WGCNA, gene significance (GS) was used to describe the relationship between gene and phenotype. Module membership (MM) was calculated to evaluate the importance of a gene in the module by using the cor function. In this study, genes with both GS > 0.3 and MM > 0.9 was defined as hub genes among the candidate gene modules (Jin et al., 2021). The correlation relationship of hub genes was explored by using the gpairs package (Emerson and Green, 2020).

### Identification of DEGs

We first used lmFit and eBayes functions in the limma package (Ritchie et al., 2015) to identify the DEGs between VaD and control groups. The statistical method to calculate false discovery rate (FDR) was the Benjamini–Hochberg method. Then a threshold of adjust-p < 0.05 and the absolute value of log_2_ fold change (log_2_FC) > 1 were set, and the significant DEGs between the VaD and controls were filtered out. A volcano plot was presented by using EnhancedVolcano (Blighe et al., 2018). The distribution shape of TLR2 was shown in the violin plot by using the ggpubr package (Kassambara, 2020).

### Geno Ontology and Kyoto Encyclopedia of Genes and Genomes Enrichment Analysis

A GO enrichment analysis was run to annotate the functions of the significant DEGs with GO terms. The GO enrichment analysis could explain the features of changed genes from the following three structural networks of terms: biological processes (BP), cellular components (CC), and molecular functions (MF). The KEGG pathway analysis was performed to investigate the pathway that the significant DEGs might be involved in. The org. Hs.eg.db package (Carlson, 2021) was used for transition from gene symbols to Entrez ID. Then the clusterProfiler package (Yu et al., 2012; Wu et al., 2021) was used for the enrichment analysis. At last, the ggplot2 (Wickham, 2016) package was used for visualization. The aforementioned analysis results enabled us to discover the biological pathways of the altered genes in the VaD group.

### Gene Set Enrichment Analysis (GSEA)

In the GSE122063 datasets, GSEA was used to explore distinct GO terms and KEGG pathways that may be associated with TLR2. All genes were included in the analysis. Gene sets were directly downloaded from the website (http://www.gsea-msigdb.org/gsea/downloads.jsp). Except for the VaD and control groups, we set the median expression level of TLR2 as the cutoff value to divide patients into TLR2-high and TLR2-low expression groups. The org. Hs.eg.db package (Carlson, 2021) was used for Entrez ID transition, and the clusterProfiler package (Yu et al., 2012; Wu et al., 2021) was used for the enrichment analysis. Furthermore, the gseaplot2 function in the enrichplot package (Yu, 2021) was used for visualization of enrichment results.

### Construction of a Protein–Protein Interaction Network

We used the STRING online database (https://string-db.org/) to construct a PPI network. Significant DEGs were uploaded to the STRING website. After being filtered by the “no more than 50 interactors” and “k-means clustering” options, the PPI network was exported into a TSV file. At last, the analysis and visualization of the interaction network were achieved by Cytoscape software. The function of network analysis function in the Cytoscape software calculated the degree which was utilized as the continuous mapping of nodes both in size and fill color (from blue to red). The combined score exported directly from the string database was used for the continuous mapping of edges both in width and stroke color (from blue to red). Larger size and bluer nodes indicated the higher degree, while wider and bluer lines indicated the higher combined scores.

## Results

### WGCNA and Module Related With VaD

**FIGURE 1:**
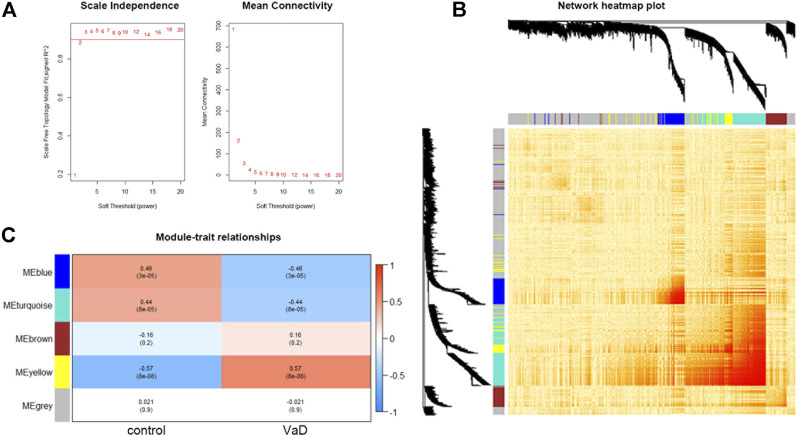
Results and visualization of Weighted Gene Co-expression Network Analysis (WGCNA) analysis. **(A)** Determination of soft threshold β. Left: scale independence; right: mean connectivity. **(B)** Heat map showing the TOM among all 5,000 genes involved in the WGCNA with cluster dendrogram showing on the axis. Each color represents one specific co-expression module; the above branches represent genes. The genes with strong correlations are clustered into modules, which are represented as dark sections symmetrically distributed along the diagonal in the heatmap, corresponding to the cluster dendrogram. **(C)** Module–trait relationships among the five gene modules. The yellow module is the most correlated module (correlation coefficient = 0.57, ***p < 0.001).

Using the expression matrix, WGCNA was used to determine the main module which was most linked with VaD. At first, we chose the top 5,000 genes sorted by MAD in the GSE122063 microarray assay for analysis. According to the calculation result, the soft-thresholding power β was 2 as the plot showed, with the scale-free topology R2-value achieving 0.9 (Figure 1A). To visualize the weighted network, a heat map was plotted. The gene co-expression network was created, and the genes were clarified into five modules represented by distinct colors including grey, turquoise, blue, brown, and yellow. This is called a cluster dendrogram, and it was presented along the axis. a network heat map of all 5,000 genes was shown by using the TOMplot function in Figure 1B. Each row and column in the heat map represented the same gene, and thus the network heat map is a symmetric plot. The genes with strong correlations were clustered into modules, which were represented as dark sections symmetrically distributed along the diagonal in the heat map, corresponding to the cluster dendrogram. The biggest grey module included 2,783 genes, and the smallest yellow module included 400 genes. As shown in the module–trait relationships plot, the yellow module was most positively associated with VaD (correlation coefficient = 0.57, ***p < 0.001; Figure 1C) and was chosen as the key module. The functional annotation of three significantly related modules (blue, turquoise, and yellow) are shown in Supplementary Figure S1. The yellow module was most related to immunity and inflammation.

### Identification of Hub Genes and Top Hub Gene

**FIGURE 2:**
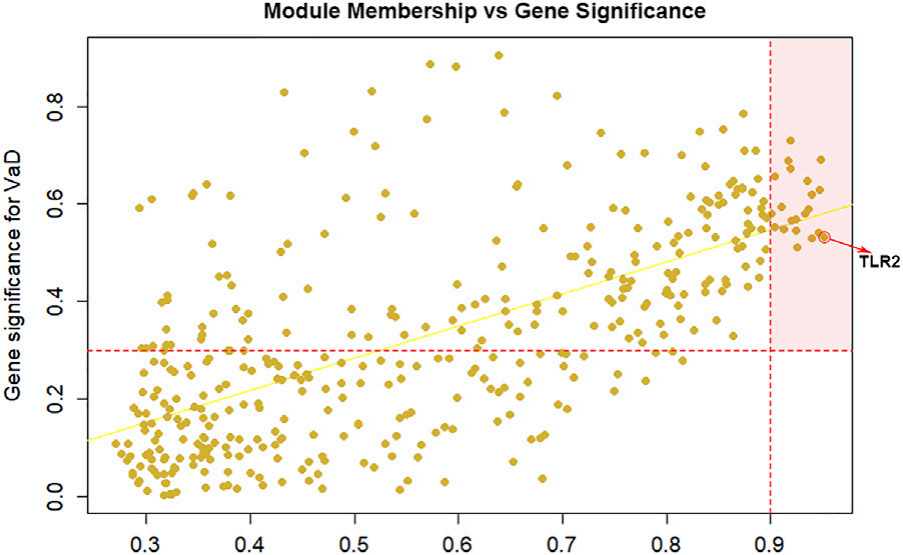
Selection of hub genes. Module membership (MM) vs. gene significance (GS) in the yellow module (correlation coefficient = 0.65, ***p < 0.001). The red dotted lines represented the thresholds of MM > 0.9 and GS > 0.3 set for hub genes and separated an area in the upper right corner. Toll-like receptor 2 (TLR2) is selected as the top hub gene.

Among the 400 genes in the yellow module, genes with MM > 0.9 and GS > 0.3 were sorted out as hub genes. The red dotted lines represent the thresholds value of MM > 0.9 and GS > 0.3 set for hub genes and separated an area in the upper right corner. The correlation analysis between yellow module memberships and gene significance showed statistical significance (correlation coefficient = 0.65, ***p < 0.001). In total, 21 hub genes were identified (TLR2, CD163, VSIG4, SLAMF8, C1QB, CD16a, CD32, ALOX5AP, integrinβ2, EBI3, HCLS1, CD14, LAIR-1, CD300a, IFI30, LCP1, C1orf162, γ-parvin, ALOX5, SLA, and CMTM7). According to MM or the chooseTopHubInEachModule function, TLR2 was the top hub gene in the yellow module (Figure 2). Furthermore, we found that TLR2 shows a strong positive correlation with other candidate genes, which indicated that changes in TLR2 expression might cause changes in these genes (Supplementary Figure S2).

### Identification of DEGs in VaD

**FIGURE 3:**
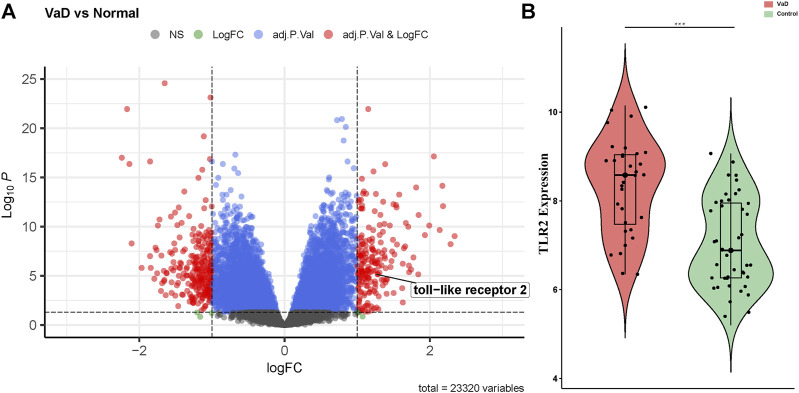
Differentially expressed genes (DEGs) present in vascular dementia (VaD) and control groups in microarray from GSE122063 and the expression level of Toll-like receptor-2 (TLR2). **(A)** Volcano plot showed the distribution of the DEGs between two groups. The red dots correspond to the significantly regulated genes. **(B)** Violin plot of TLR2. TLR2 is upregulated in the VaD group (***p < 0.001).

The gene expression levels of the samples were distributed at the same baseline after normalization. Compared to the control group, significant DEGs were identified in the VaD group by setting the threshold value as adjust-p < 0.05 and |log2FC| > 1. The expression of the genes was displayed as a volcano plot in which the size of the dot reflects |log2FC| of the gene (Figure 3A). There were 456 significant DEGs between the VaD and control groups among the 23,320 genes detected in microarray, including 198 upregulated ones and 258 downregulated ones. TLR2 was one of the significant DEGs and was marked out in the volcano plot. Specifically, the expression level of TLR2 in the VaD and control groups was shown in the violin plot (***p < 0.001, Figure 3B). TLR2 was significantly differentially expressed between the two groups.

### Results of GO and KEGG Analysis

**FIGURE 4:**
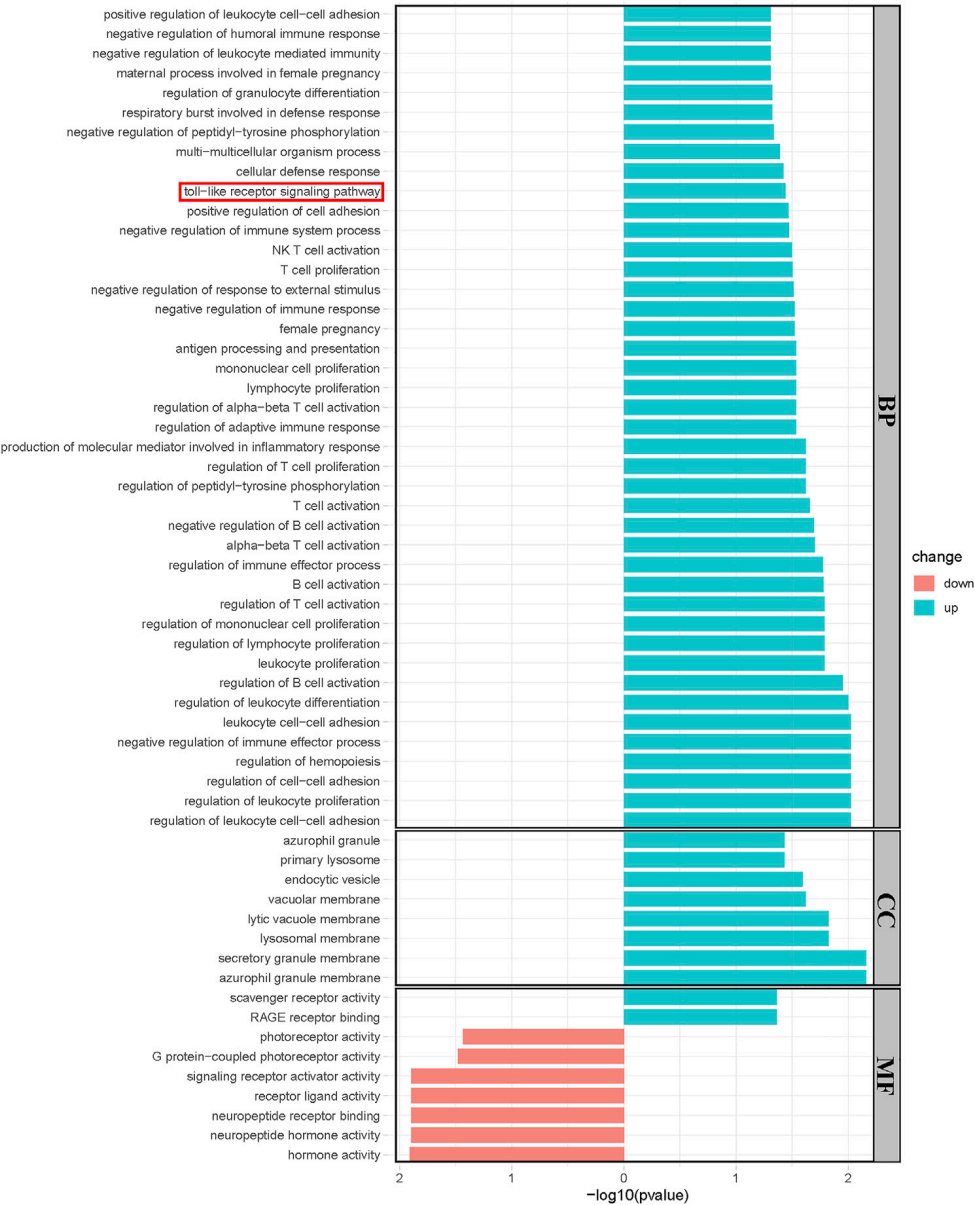
Results of the Geno Ontology (GO) terms enrichment analysis of significant DEGs. (*p < 0.05). Blue bars showed the results of upregulated genes while red bars showed the results of downregulated genes.

Significantly upregulated and downregulated DEGs were enriched in BP, CC, and MF terms and the KEGG pathway, respectively. The horizontal axis represents −log10 (*p*-value), while the color indicated the change direction. In detail, BP, Toll-like receptor signaling pathway was enriched, which was consistent with our previous result. Other BPs such as negative regulation of immune system process, antigen processing, and presentation and regulation of B, T, and NK cells were examples of significantly enriched upregulated GO terms (*p < 0.05, Figure 4A), while CCs, including azurophil granule, endocytic vesicle, and secretory granule membrane are shown (*p < 0.05, Figure 4B). Upregulated MFs, such as scavenger receptor activity and RAGE receptor activity, were significantly enriched. Neuropeptide hormone activity, neuropeptide receptor binding, and signaling receptor activation activity were downregulated (*p < 0.05, Figure 4C). Most enriched KEGG pathways did not reach statistical significance in which we observed a trend in Toll-like receptor signaling pathway and neuroactive ligand–receptor interaction was significantly downregulated (Supplementary Figure S3).

### GSEA Enrichment Results

**FIGURE 5:**
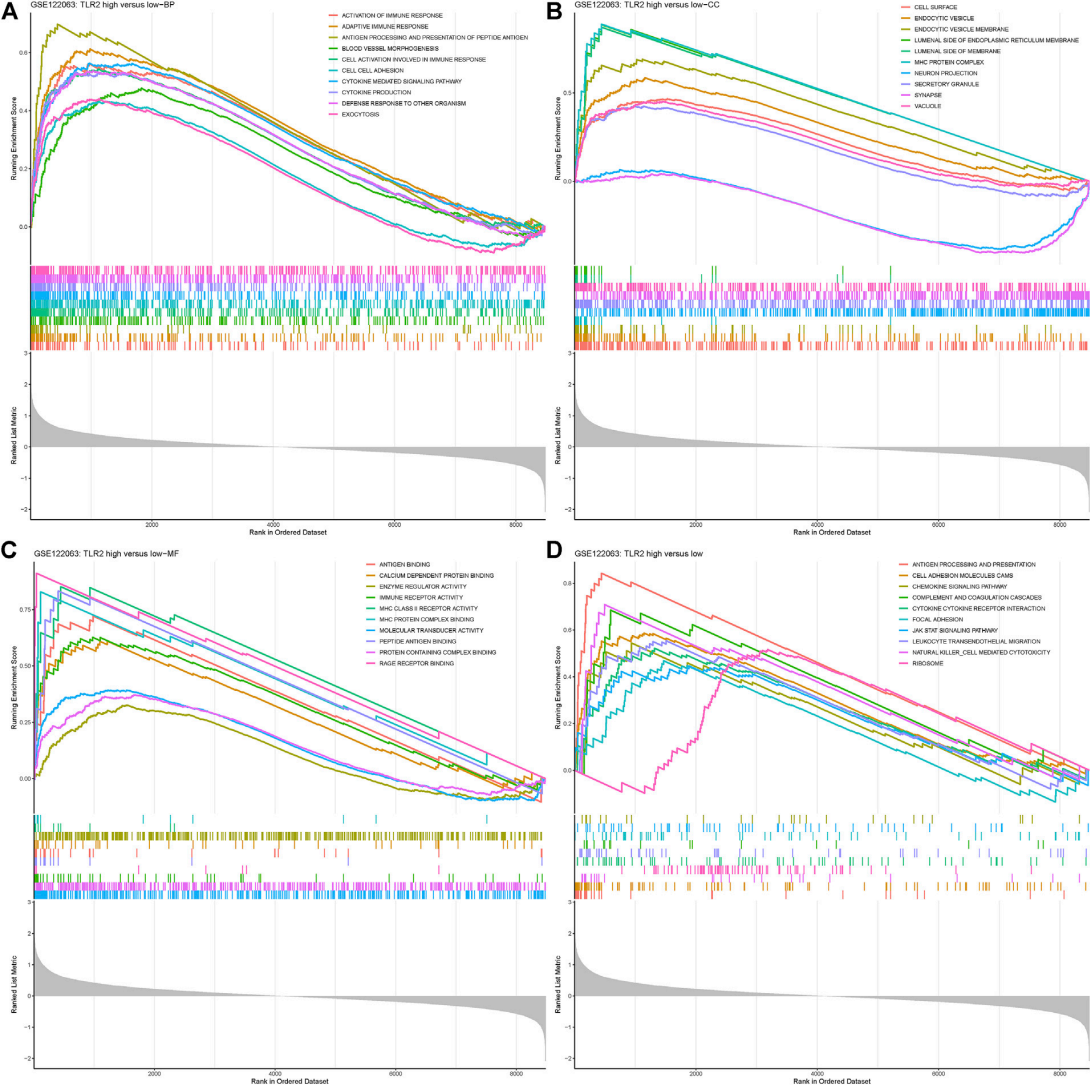
Gene Set Enrichment Analysis (GSEA) results grouped by the expression level of TLR2. **(A)** BP enriched in TLR2-high group. **(B)** CC enriched in TLR2-high group. **(C)** MF enriched in TLR2-high group. **(D)** KEGG pathways enriched in TLR2-high group.

GSEA was analyzed in the disease group versus control as well as groups divided by the expression level of TLR2. When comparing the VaD group with the control group, the Toll-like receptor pathway was enriched, which was the same as the results from DEGs. Other immunity and inflammation-related processes were also enriched which indicated the representativeness of the data and complemented evidence for the role of TLR2 in neuroinflammation. The results are shown in Supplementary Figure S4. When comparing the TLR2-high group with the low group, the results showed that BPs, such as cytokine-mediated signaling pathway and defense response to other organism, were significantly enriched in the TLR2-high samples (*p < 0.05, Figure 5A). CCs, such as synapse, vacuole, and cell surface granule, and MFs, such as immune receptor activity and molecular transducer activity, were significantly enriched in the TLR2-high samples, shown in Figures 5B, C, respectively (*p < 0.05, Figures 5B,C). When it comes to the KEGG enrichment analysis, pathways such as antigen processing and presentation, ribosome, and cytokine–cytokine receptor reaction were significantly over-represented in TLR2-high samples (*p < 0.05, Figure 5D). The similar enrichment results in VaD and control groups, as well as in the TLR2-high and low groups further demonstrated the important role of TLR2 in VaD. Moreover, high expression level of TLR2 was related to many genes, including myeloid differentiation factor 88 (MyD88), nuclear factor kappa B (NF-κB), protein kinase B (AKT), glial fibrillary acidic protein (GFAP), ionized calcium-binding adapter molecule 1 (Iba1), and many cytokines according to the expression matrix and the KEGG pathway.

### PPI Network Construction

**FIGURE 6:**
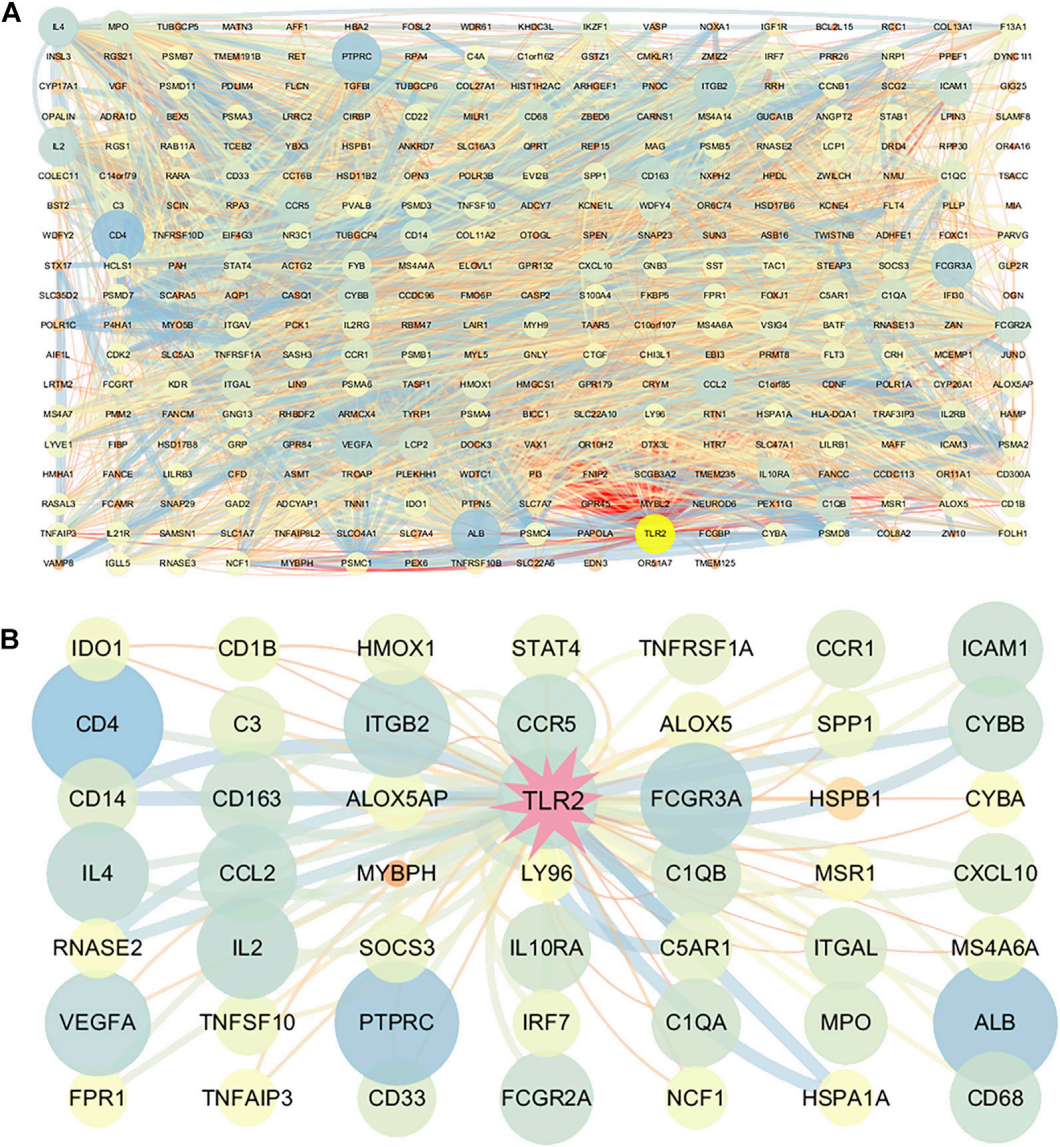
Construction of the protein–protein interaction (PPI) network consisting of DEGs. **(A)** PPI of DEGs. **(B)** Partial network centered on TLR2. The size and color of the nodes reflect the degree and the width and color of the edges reflect the combined scores (color: from blue to red). Larger size and bluer nodes indicated the higher degree while wider and bluer lines indicated the higher combined scores.

With the combined use of STRING and Cytoscape, the PPI network of the significant DEGs was created. The size and color reflected the degree of nodes in which the more edges connected to this node, the greater its degree. The larger size and bluer node indicated the higher degree. The width and color reflected the combined score of edges in which the combined scores were positively related to the interaction relationships between the two proteins. The wider and bluer line indicated higher combined scores. The overall network of DEG-correlated proteins is shown in Figure 6A. TLR2 got a relatively high degree in this overall network which suggested that TLR2 played a crucial role in the network. Considering the complication of the network, a new network centered on TLR2 was further constructed and amplified. TLR2 was most associated with Complement C5a Receptor 1 (C5AR1), Heat Shock Protein Family A Member 1 A (HSPA1A), cluster of differentiation (CD14), and cytochrome B-245 Beta Chain (CYBB) (Figure 6B).

## Discussion

Cognitive impairment related to aging has become one of the major public health burdens for us. Although Alzheimer’s disease is the most prevalent cause of clinically diagnosed dementia in western nations, vascular etiology is the second most common cause. Also, vascular etiology is the most common cause in East Asia (Iadecola et al., 2019). Thus, it is worthwhile to investigate the underlying mechanism of VaD development. Much progress has been made during the past years; however, several controversies remain to be interpreted.

In the present study, we first took use of WGCNA to achieve modules related to VaD and hub genes. According to the correlation coefficient, a yellow module was chosen which was closely related to immunity and we finally identified 21 hub genes. TLR2 was the top hub gene which was strongly correlated with other possible candidate genes. Second, we filtered out 456 significant DEGs by adjust-*p* < 0.05 and |log2FC| > 1. TLR2 was one of the DEGs and was significantly upregulated in the VaD group. Third, significantly upregulated and downregulated DEGs were gone through GO and KEGG analyses and the Toll-like receptor pathway, and other inflammation related processes were found to be upregulated in the VaD group. Fourth, GSEA results showed that cytokine-mediated signaling pathway, cell surface, immune receptor activity, and cytokine–cytokine receptor reaction were significantly over-represented in TLR2-high samples. The results were similar to enrichment results achieved by samples being divided by disease status. Finally, in the PPI analysis, TLR2 was an important node with a higher degree and combined scores edges which indicated that TLR2 remained a key target at the protein level. In summary, with five approaches complementing each other, TLR2 might participate in the pathophysiological process of VaD *via* the neuroinflammation pathway.

**FIGURE 7:**
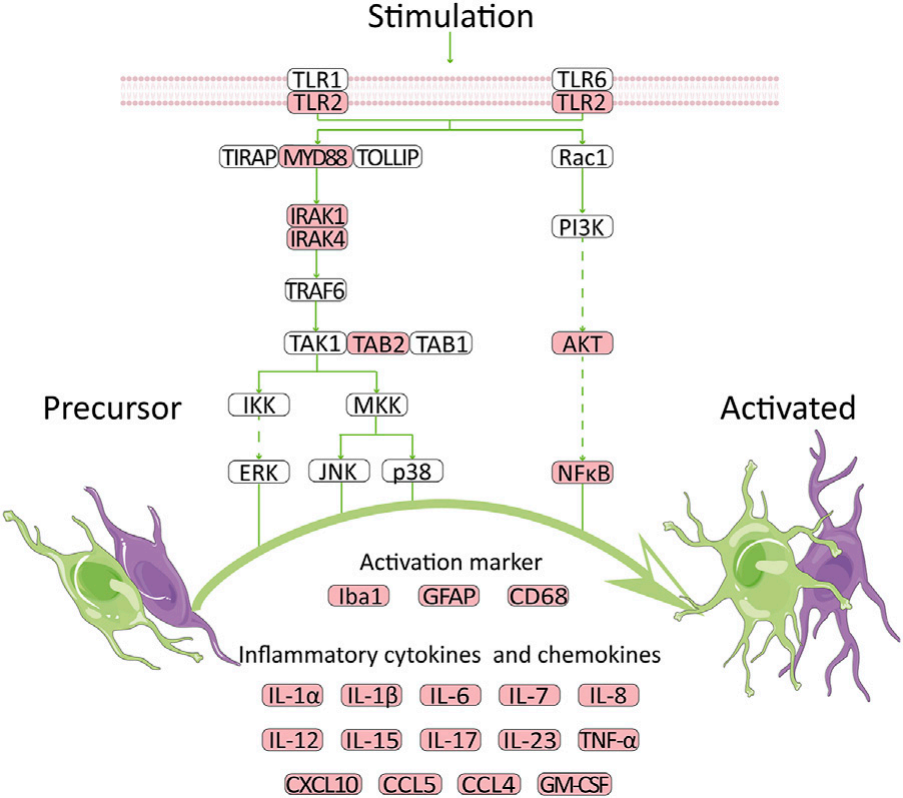
Potential mechanism for high expression of TLR2 to promote VaD. The network is summarized according to GSE122063 database and public KEGG pathway. Red indicates the upregulated genes.

TLRs were proved to be involved in the control of immunity and neurological diseases (Racke and Drew, 2009; Kouli et al., 2019; Lin et al., 2019; Tajalli-Nezhad et al., 2019; Fitzgerald and Kagan, 2020). TLR2, as a member of TLRs, also played a vital role in nervous system. Based on the KEGG Toll-like receptor signaling pathway, we summarized a mechanism chart. After comparing the pathway with our analysis results, we found that a high expression level of TLR2 was related to many genes, including MYD88, AKT, NF-κB, Iba1, GFAP, and many cytokines, suggesting that TLR2 might participate in the development of VaD *via* the neuroinflammation pathway. The genes that were upregulated in this microarray were marked in red. High expression of TLR2 induced activation of astrocytes and microglia, which further lead to the secretion of cytokines (Figure 7). Previous studies were consistent with our results and provided a foundation for this prediction. Knockdown of MyD88 attenuated the mRNA expression of TNF-α and inducible nitric oxide synthase (iNOS) (Jana et al., 2008) in AD, while reduced inflammatory response was observed in MYD88 knockdown mice with traumatic brain injury (TBI) (Krieg et al., 2017). These results revealed the role of MYD88 in neuroinflammation. Meanwhile, AKT and NF-κB were involved in the neuroinflammation pathway in experimental models of AD (Yang et al., 2020). In addition, GFAP is an activation marker of astrocytes, while Iba1 and CD68 are the activation markers of microglia. The anti-TLR2 antibody group had lower GFAP and CD68 immunoreactivity than the control group (McDonald et al., 2016). At last, the expression levels of inflammatory cytokines increased (Brea et al., 2011; Dzamko et al., 2017; Sun et al., 2017). At the protein level, TLR2 was proven to be strongly correlated with proteins such as C5AR1 (Mödinger et al., 2018), HSPA1A (Yang et al., 2013), and CD14 (Aguilar-Briseño et al., 2020), according to the previous study which was coincident with our results. These molecules, as well as CYBB, were all related to neuroinflammation which further proved our results (Tarassishin et al., 2014; Qu et al., 2017; Michailidou et al., 2018; Keller et al., 2021). All the results proved that TLR2 could be an efficient target to regulate the unwanted inflammatory responses in neurological conditions (Hayward and Lee, 2014). Thus, we suggested that TLR2 might participate in the development of VaD *via* the neuroinflammation pathway.

In parallel, there was other evidence that also supported the role of TLR2 in the development of VaD. First, TLR2 regulated the risk factors of vascular diseases which further affect VaD development, such as atherosclerosis (Li B et al., 2020) and diabetes. TLR2 was found to promote vascular smooth muscle cell chondrogenic differentiation and consequent calcification in atherosclerosis by activating p38 and extracellular regulated protein kinases (ERK) 1/2 signaling (Lee et al., 2019). Activation of TLR2 stimulated the pro-inflammatory cytokines and chemokines secretion, which would cause vascular injuries. Diabetes-induced changes in cerebral blood flow and cognitive deficits were prevented when TLR2 was knocked out (Hardigan et al., 2017).

Second, TLR2 participated in the pathophysiological process of stroke and other neurodegeneration diseases. In IS, TLR2 was associated with the outcome (Brea et al., 2011), and TLR2 inhibition improved neuronal survival (Ziegler et al., 2011), which indicated a future therapy. Repeated exposure to TLR2 agonists may exacerbate neurodegeneration in AD by their microglial-mediated toxicity (Lax et al., 2020) and inhibition of TLR2 in microglia (Liu et al., 2012) or mouse model could be beneficial in AD pathogenesis. Similarly, TLR2 was reported to exert a prominent role in the microglial-mediated responses which is vital for PD progression (Doorn et al., 2014).

Third, TLR2 exerted functions in biological processes or other neurological diseases *via* the neuroinflammation pathway. Neuraminidase-induced inflammatory reaction *in vivo* was partly dependent on TLR2 (Fernández-Arjona et al., 2019), while interferon-γ (IFN-γ) enhanced α-syn stimulation and inflammatory responses *via* TLR2, TLR3, and TNF-α *in vitro* (Wang et al., 2019). TLR2 and TLR4 could serve as important mediators of repeated social defeat stress (R-SDS)–induced microglial activation in the medial prefrontal cortex (mPFC), which caused neuronal and behavioral alternations *via* inflammatory-related cytokines (Nie et al., 2018). In addition, TLR2 and TLR4 were shown to potentially advance secondary brain injury after experimentally controlled cortical impact (CCI) *via* neuroinflammation (Krieg et al., 2017) while activation of microglia, *via* a TLR2-sphingosine kinase 1 (Sphk1)-pro-inflammatory cytokines (IL-1β, TNF-α, IL-17, and IL-23) pathway, may be involved in ischemia/reperfusion (I/R) injury (Sun et al., 2017). In IS, TLR2 activation was associated with a higher interleukin (IL)-1β, tumor necrosis factor-α (TNF-α) and IL-6 expression level (Brea et al., 2011). The expression of TLR2 was increased in affected regions, further inducing TNF-α expression and increased phosphorylation of NF-κB p105 subunit in PD (32). In AD, TLR2 was proved to be a natural receptor for Aβ to trigger neuroinflammatory activation (Richard et al., 2008; Liu et al., 2012). TLR2 deficits in microglia shifted related inflammatory activation *in vivo,* while TLR2 insufficiency reduced Aβ42-triggered inflammatory activation and increased Aβ phagocytosis *in vitro*, which were both related to improved neuronal function (Jana et al., 2008; Liu et al., 2012; McDonald et al., 2016). TLR2 could enhance macrophage receptor with collagenous structure (Marco)–induced neuroinflammation by acting on the scavenger receptors cysteine-reach (SRCR) domain of Marco, which also suggested that TLR2 could serve as a novel target for reducing neuroinflammation in neurodegenerative diseases (Wang et al., 2021). Therefore, it is reasonable to speculate that TLR2 participates in the pathophysiological process of VaD through the neuroinflammation pathway and could serve as a key target.

Our research showed that using bioinformatics to investigate the molecular processes underlying VaD could provide valuable information. Bioinformatic techniques, however, were used to identify probable critical pathways and genes. Thus, molecular experiments based on clinical samples or animal models should be performed to further validate the results. It remained to be clarified whether TLR2 is involved in the pathophysiological process of VaD and inhibition of TLR2 would contribute to VaD treatment.

In conclusion, we identified TLR2 as a neuroinflammatory leading change during VaD.

## Data Availability

The datasets presented in this study can be found in online repositories. The names of the repository/repositories and accession number(s) can be found at: https://www.ncbi.nlm.nih.gov/geo/, GSE122063.
