# Supplementary figures and images for: Identification of TLR2 as a Key Target in Neuroinflammation in Vascular Dementia
Source: Front Genet. 2022 Jul 6;13:860122. doi: 10.3389/fgene.2022.860122 (PMC9296774; doi:10.3389/fgene.2022.860122)

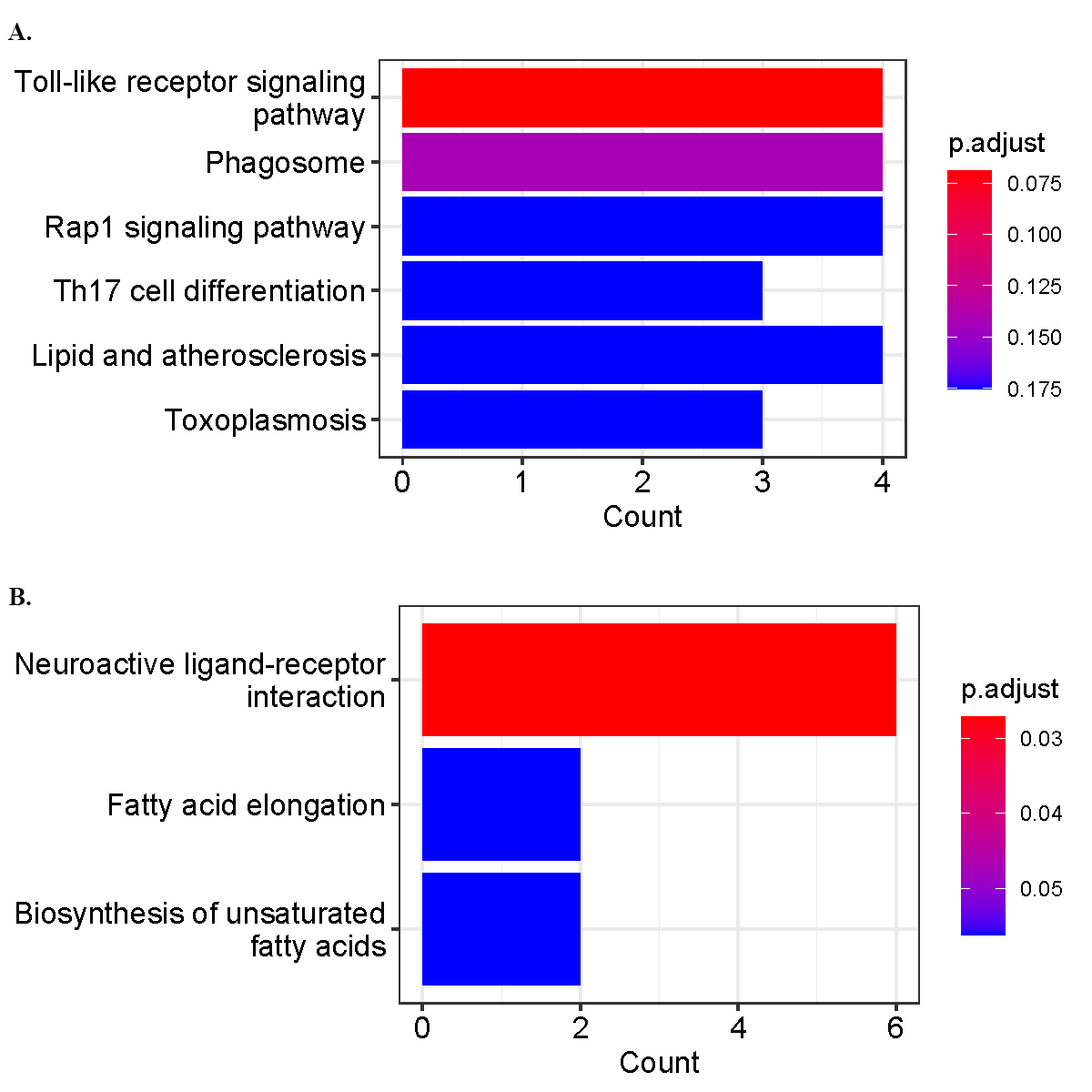

Supplement: Supplementary file 1 [file Image3.TIF]

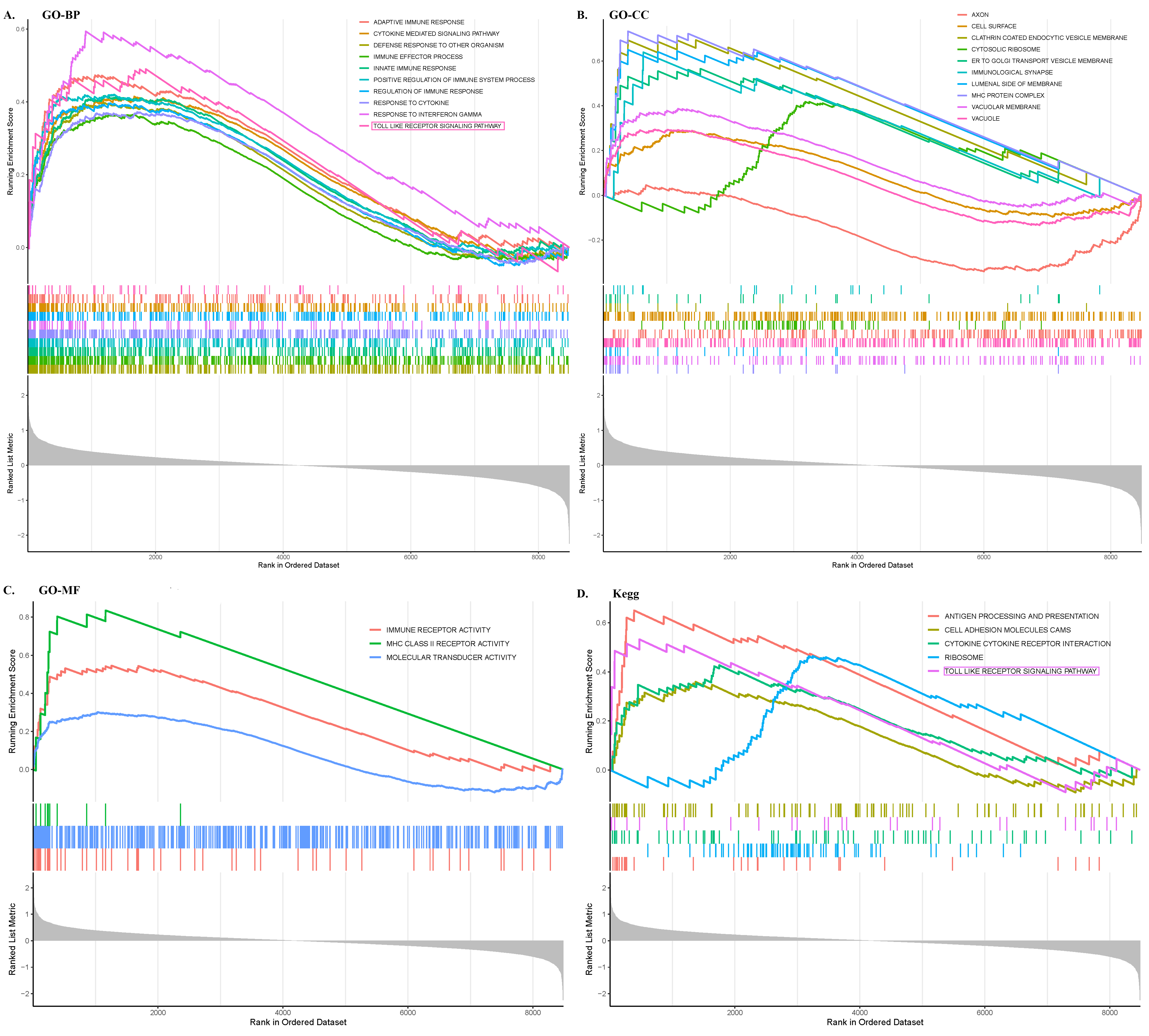

Supplement: Supplementary file 2 [file Image4.TIF]

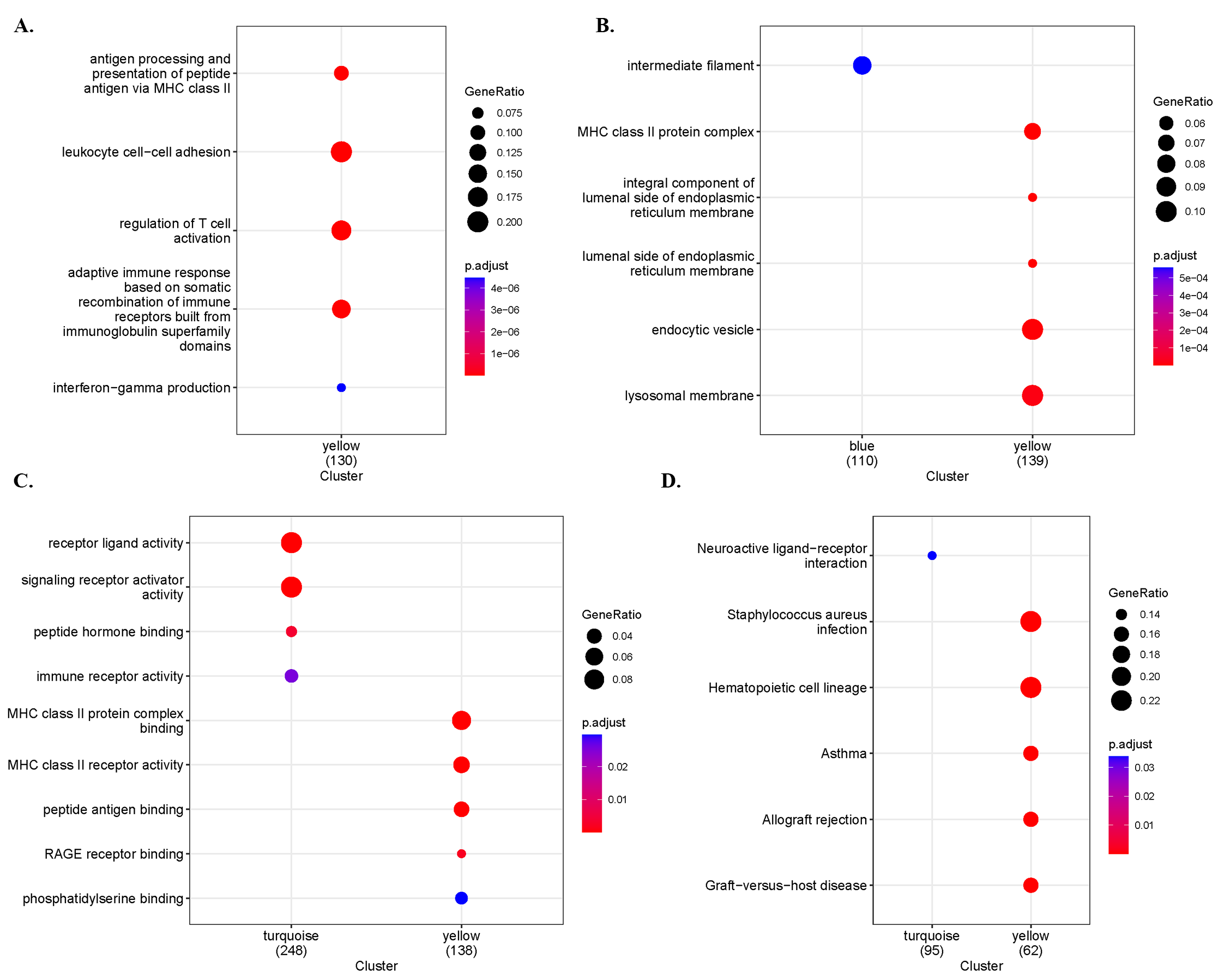

Supplement: Supplementary file 3 [file Image1.TIF]

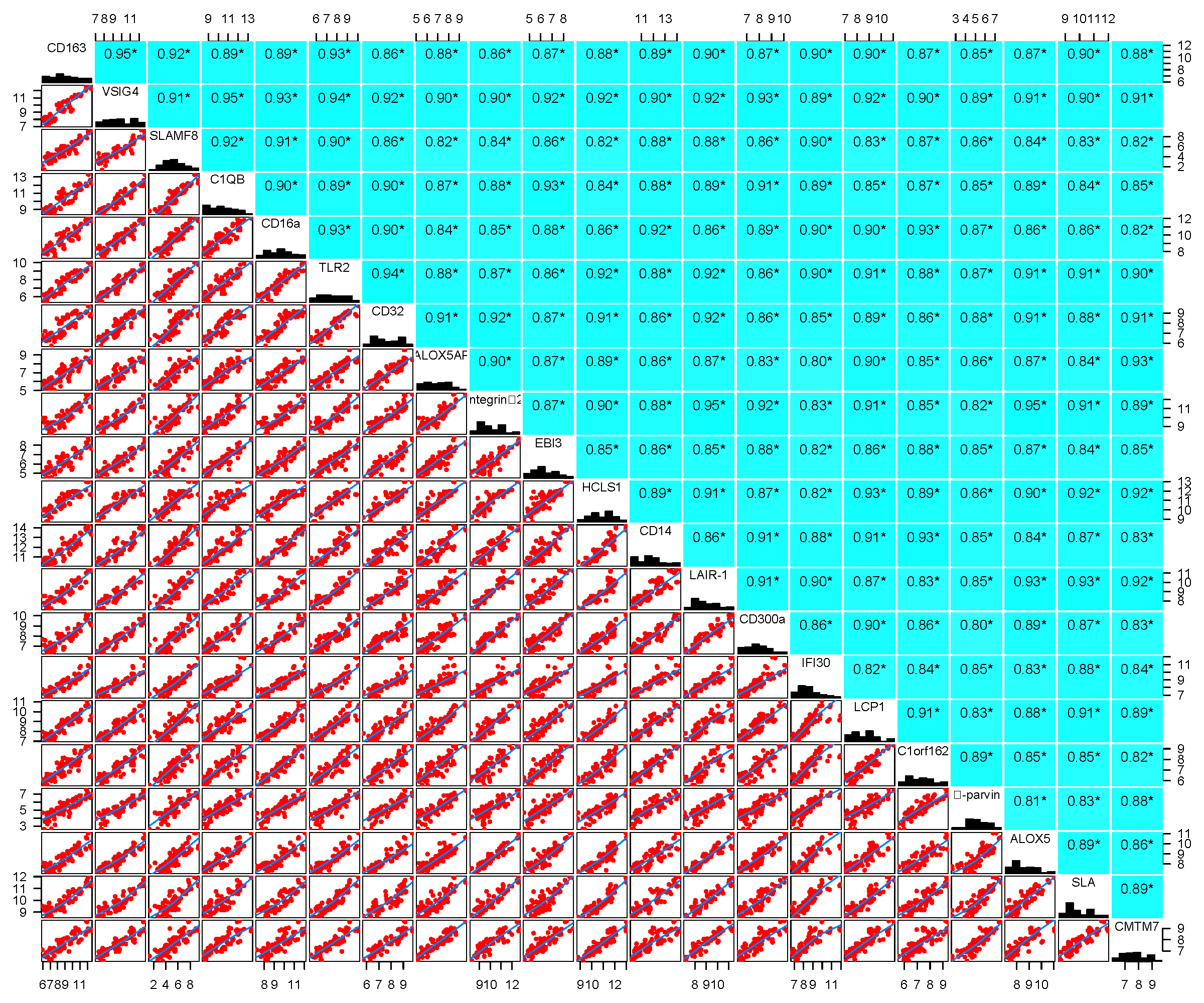

Supplement: Supplementary file 4 [file Image2.TIFF]
